# Prospect of making XPS a high-throughput analytical method illustrated for a Cu_*x*_Ni_1−*x*_O_*y*_ combinatorial material library†

**DOI:** 10.1039/d1ra09208a

**Published:** 2022-03-11

**Authors:** Lucas C. W. Bodenstein-Dresler, Adi Kama, Johannes Frisch, Claudia Hartmann, Anat Itzhak, Regan G. Wilks, David Cahen, Marcus Bär

**Affiliations:** Dept. Interface Design, Helmholtz-Zentrum Berlin für Materialien und Energie GmbH Berlin Germany lucas.bodenstein-dresler@helmholtz-berlin.de lucas.bodenstein-dresler@tu-dortmund.de; Bar-Ilan Inst. for Nanotechn. & Adv. Materials, BINA, Dept. of Chemistry, Bar-Ilan University Ramat Gan Israel 5290002; Energy Materials In-Situ Laboratory Berlin (EMIL), Helmholtz-Zentrum Berlin für Materialien und Energie GmbH Berlin Germany; Dept. of Mol. Chemistry and Materials Sci., Weizmann Institute of Science Rehovot Israel 7610001; Department of Chemistry and Pharmacy, Friedrich-Alexander-Universität Erlangen-Nürnberg Erlangen Germany; Helmholtz-Institute Erlangen-Nürnberg for Renewable Energy (HI ERN) Berlin Germany

## Abstract

Combinatorial material science crucially depends on robust, high-throughput characterization methods. While X-ray photoelectron spectroscopy (XPS) may provide detailed information about chemical and electronic properties, it is a time-consuming technique and, therefore, is not viewed as a high-throughput method. Here we present preliminary XPS data of 169 measurement spots on a combinatorial 72 × 72 cm^2^ Cu_*x*_Ni_1−*x*_O_*y*_ compositional library to explore how characterization and evaluation routines can be optimized to improve throughput in XPS for combinatorial studies. In particular, two quantification approaches are compared. We find that a simple *integration* (of XPS peak regions) approach is suited for fast evaluation of, in the example system, the [Cu]/([Cu] + [Ni]) ratio. Complementary to that, the time-consuming (XPS peak-) *fit* approach provides additional insights into chemical speciation and oxidation state changes, without a large deviation of the [Cu]/([Cu] + [Ni]) ratio. This insight suggests exploiting the fast *integration* approach for ‘real time’ analysis during XPS data collection, paving the way for an ‘on-the-fly’ selection of points of interest (*i.e.*, areas on the sample where sudden composition changes have been identified) for detailed XPS characterization. Together with the envisioned improvements when going from laboratory to synchrotron-based excitation sources, this will shorten the analysis time sufficiently for XPS to become a realistic characterization option for combinatorial material science.

## Abstract 2

Methods for fast quantification of XPS data of a Cu_*x*_Ni_1−*x*_O_*y*_ combinatorial material library were evaluated in a step towards high-throughput analysis.

## Graphical abstract

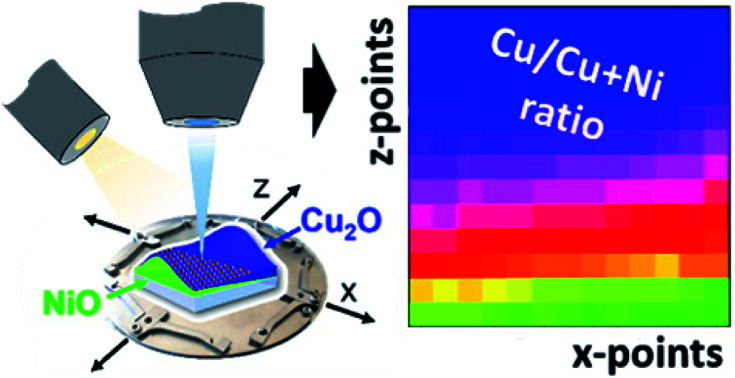

## Introductions

Combinatorial material science allows screening of large compositional spaces for desired functional properties to discover new materials, for example for device applications. Combinatorial material libraries, like the one we are investigating in this study, can be described as a single substrate holding many experiments.^1^ This definition can be realized by creating intentional gradients in composition and/or thickness across the substrate. For example, over 5000 possible compositions can be produced by combining three elements with composition step changes of 10 at % for each element (for more details, see S.I.†). These libraries are usually deposited on relatively large – compared to usual laboratory-scale – substrates to increase the number of (accessible) experiments per library. Even though this approach can reduce the material costs and deposition time, the increased number of samples/experiments on a single library comes with an enormous expansion of times for measurement and data evaluation, motivating the development of (semi)automated methods. Therefore, high-throughput methodologies, which balance the requirements for measuring robust and relevant material properties with rapid characterization, are needed for efficient combinatorial materials research,^2^ explaining the focus on so called standard measurement techniques so far. However, advanced analytics can provide information that is not available from the standard techniques. Thus, X-ray photoelectron spectroscopy (XPS), the method highlighted in our study, provides information about the chemical and electronic (surface) structure of materials that present the initial situation from where the interface properties develop in thin-film stacks of modern (opto)electronic devices. In addition to the elemental (surface) composition, XPS can identify chemical species. This insight cannot be (easily) accessed by standard measurement techniques, but is a prerequisite to expedite deliberate device-driven new material discoveries. However, data acquisition and evaluation of classical XPS are time-consuming depending on the measured energy range, needed energy resolution, and numbers of sweeps required to collect high-quality data. Thus, using XPS in combinatorial materials research has been limited.^3,4^

The results of the present study illustrate the feasibility of XPS data collection and analysis in combinatorial materials research, by testing the prospects for establishing XPS as a high-throughput characterization tool. The system chosen is a combinatorial oxide library with a compositional spread of Cu and Ni, *i.e.*, mostly comprising double metal oxide (MO) material.

Cu_*x*_Ni_1−*x*_O_*y*_ is potentially useful as a hole-selective transport layer (HTL) for halide perovskite (HaP) absorbers in solar cells. HaP-based cells have shown unprecedented performance evolution over the past decade, currently reaching power conversion efficiencies exceeding 25%.^5^ NiO has a 3.7 eV direct bandgap, a hole mobility of ≈3 cm^2^ V^−1^ s^−1^, and weak optical absorption in the visible wavelength range.^6^ Cu_2_O has a smaller bandgap (2.1–2.3 eV) with also weak visible light absorption, but with higher hole mobility, >100 cm^2^ V s^−1^.^7,8^ Combining both binary oxides promises to open a route to tune optoelectronic and structural properties to arrive at a new material, optimized for hole-conduction, electron-blocking^9,10^ with ideal interface energetics^11^ as HTL in HaP-based optoelectronic devices.

## Experimental

### Synthesis

The Cu_*x*_Ni_1−*x*_O_*y*_ combinatorial material library was deposited by pulsed laser deposition (PLD). The commercially available fluorine-doped SnO_2_ (FTO) coated glass substrate (72 × 72 mm^2^) was washed with deionized water, cleaned in an ultrasonic bath with soap, rinsed with ethanol, and washed again with deionized water. Subsequently, the washed glass substrate was treated using an Ar Plasma (PLASMA-PREEN II-862, Plasmatic Systems, Inc.) for 4 min. The substrate was then placed in the PLD chamber (Neocera) together with the target materials Cu_2_O (99.9% pure, Kurt J. Lesker Company) and NiO (99.9% pure, Kurt J. Lesker Company) and pumped down to a base pressure of 9 × 10^−5^ mbar before starting the deposition. The target-substrate distance was kept at 70 mm, the temperature of the substrate was set to 500 °C, and Ar gas was flowed into the chamber, resulting in a deposition pressure of 5 × 10^−1^ mbar. Laser pulse for ablation was done using KrF excimer laser (248 nm, CompexPro, Coherent). To realize a Cu–Ni compositional gradient of the MO film over the whole substrate, first Cu_2_O was ablated with 50 pulses on one side of the sample; then the substrate was rotated by 180°, and NiO was ablated with 50 laser pulses across from the Cu_2_O deposition sample position. This sequence was repeated 600 times for a total of 30 000 pulses per target and sample position. The laser energy density was tuned to 2.13 J cm^−2^, with a beam spot size of 0.033 cm^2^, and a repetition rate of 8 Hz. Binary Cu_2_O and NiO samples were deposited using the same setup, parameters, and glass substrates, without target and sample position alteration for reference, *i.e.*, each target was ablated separately for 30 000 pulses, on two different substrates. After deposition, the sample was taken out in a nitrogen (N_2_) flushed glove bag attached to the PLD chamber and sealed in N_2_-filled vacuum bags for transport from the Bar-Ilan University (BIU), to the Helmholtz-Zentrum Berlin für Materialien und Energie GmbH (HZB). There, the samples were introduced into a N_2_-purged glovebox directly attached to the (ultra-high vacuum) UHV-backbone of the Energy Materials In Situ Laboratory Berlin (EMIL), which can handle samples of up to 6 in diameter or 100 × 100 mm^2^ in size.^12^ The 72 × 72 mm^2^ Cu_*x*_Ni_1−*x*_O_*y*_ material library was mounted in N_2_-atmosphere on a custom-made sample holder and transferred into the UHV system to the XPS surface analysis system.

### Characterization

**Fig. 1:**
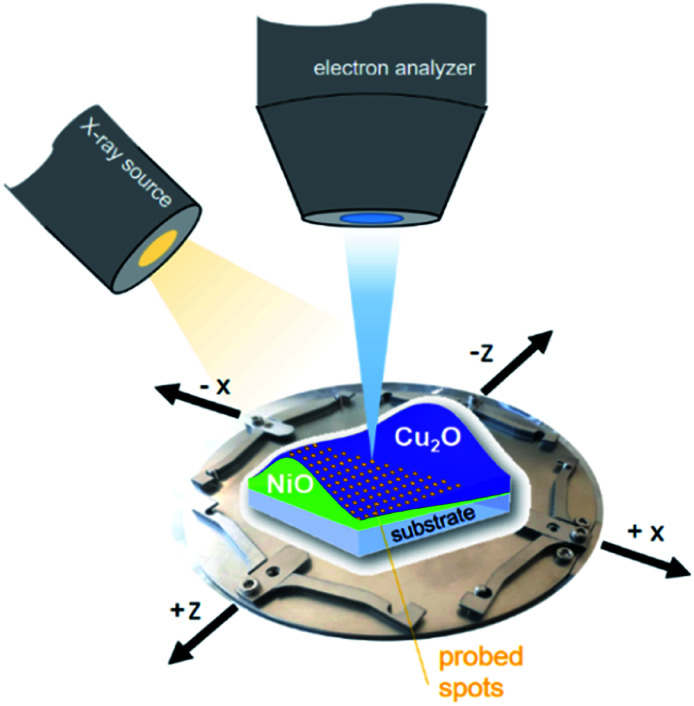
Scheme of the material library mounted on a customized sample holder, visualizing the measurement setup.

XPS measurements of the combinatorial materials library and corresponding reference samples were performed in EMIL using the surface analysis system employing a non-monochromatized PREVAC RS40B1 Mg K_α_/Al K_α_ twin anode X-ray source and a ScientaOmicron Argus CU electron analyser. The samples were studied with Al K_α_ excitation at a pressure of <5 × 10^−8^ mbar. The energy scale was calibrated using a clean Au foil, setting the Au 4f_7/2_ peak to binding energy (BE) of 84.00 eV. The measurement time to acquire the most prominent core level spectra of all elements, Ni (Ni 2p), Cu (Cu 2p), O (O 1s), with a pass energy of 30 eV and sufficient signal-to-noise ratio, was around two hours per spot. The (full width at half maxima) area illuminated by the twin anode X-ray source in that setup (see Fig. 1) was ≈2 cm^2^.^13^ The required spatial resolution was realized by selecting the aperture of the Argus CU analyser (A4) to limit the field of view to ≈4 mm^2^. In that way, the 72 × 72 mm^2^ library sample could sensibly be divided into 13 × 13 (in total 169) different measurement spots with a centre distance of 5 mm between each.

After XPS characterization a slight change of sample colour (from transparent to brownish) was observed. Thus, it cannot be excluded that the extended exposure of the Cu_*x*_Ni_1−*x*_O_*y*_ library to X-rays (in total: several 100 hours) altered some of its properties. However, no reduction or ‘metallization’, as reported for other metal oxides like WO_3_ ^14^ and MoO_3_,^15^ was observed during the measurements. In contrast, the Cu 2p data of measurement spots 7 and 163 (shown in S.I. Fig. 1a and b†) which were collected with a sample illumination time difference of approximately 100 hours, show no ‘metallization’ but do display a higher Cu(ii)/Cu(i) ratio for the sample spot that was illuminated longer.

**Fig. 2:**
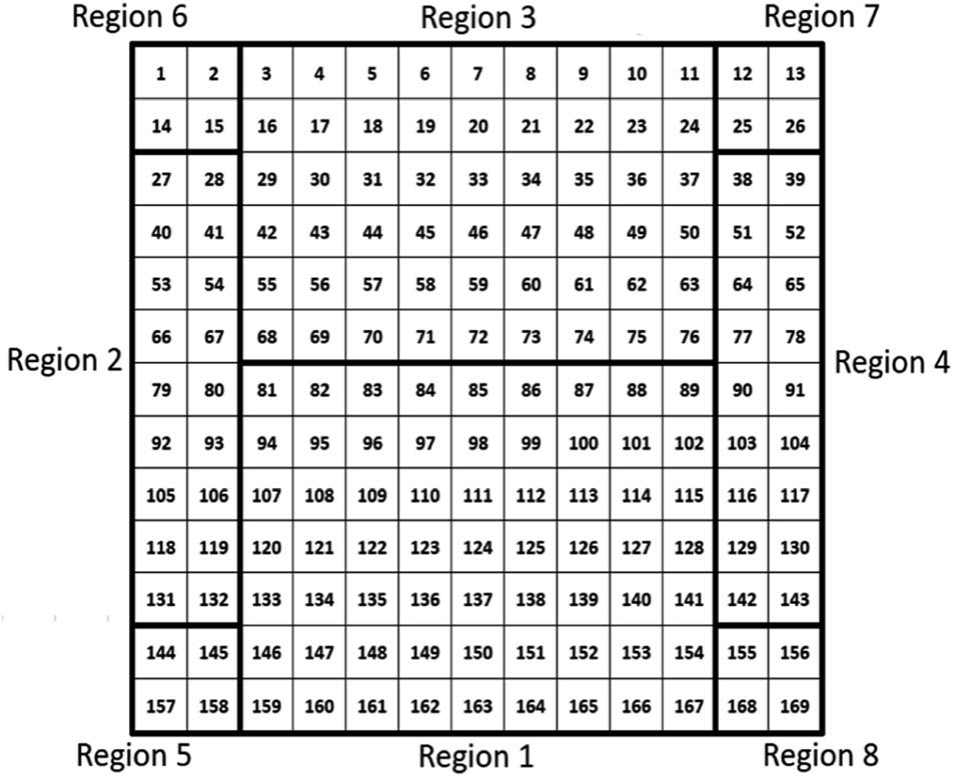
Schematic presentation of the 72 × 72 mm^2^ material library with the 169 different measurement spots that are divided into 8 different measurement regions.

When characterizing libraries, location of the measurement and moving along the sample reproducibly to a specific position is critical. This is assured by a computer-controlled stepping motor-equipped manipulator and combination with the custom-made sample holder, specifically designed for the 72 × 72 mm^2^ sample (see Fig. 1). In this way, the library can be moved in three dimensions and rotated by 360° in the *X*–*Z*-plane with highly reproducible positions given in mm for *X*-, *Y*- and *Z*-direction. Due to space constraints in the analysis chamber, the library was subdivided into eight separate measurement regions (Fig. 2; see S.I. for more details†).

Two different approaches were used to quantify the XPS data. The first (coarse) one is based on determining the area under the photoemission peaks by fast *integration*. To do so, two steps are needed before the integration. First, the K_α_-satellite peaks^16^ are subtracted from each spectrum – a process that can be easily scripted/automated. Then a linear background is subtracted from the measured Ni and Cu 2p core-level spectra. The Ni and Cu 2p intensities (*I*^integration^_Ni_, *I*^integration^_Cu_) are derived by integrating the background- and satellite-subtracted spectra in intervals of *I*_Ni_ = {852–889.75} eV and *I*_Cu_ = {924–970} eV for each of the 169 probed spots. The elemental composition – Cu content (Cu_%_ = [Cu]/([Cu] + [Ni])) – was then determined according to1
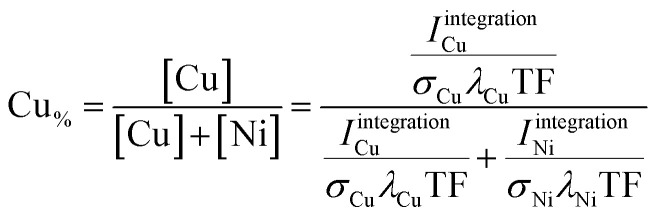
with *λ*_Cu_ and *λ*_Ni_ representing the inelastic mean free paths of the Ni and Cu 2p photoelectrons in the Cu_*x*_Ni_1−*x*_O_*y*_ material, approximated with the *λ* values of (Cu_2_O)_*x*_(NiO)_1−*x*_ (see Table S.I. 2 and related discussion for more details†). *σ*_Cu_ and *σ*_Ni_ being the element-specific photoionization cross-sections of Cu 2p and Ni 2p, respectively^17^ (*σ*_tot_ in Table S.I. 3†), and TF the analyzer transmission function for these photoelectrons (Table S.I. 3†). Even with the best attempts to adjust for the impact of different *λ* and *σ* values on the derived intensities, these corrections have significant uncertainties. Thus, we focus on relative considerations rather than on absolute values for our analyses in the following.

The second, more refined evaluation approach, involves detailed *fitting* of the Cu 2p and Ni 2p core levels, after properly accounting for satellite peaks, to differentiate between different elemental species. Before fitting, the K_α_-satellite peaks are subtracted from each spectrum and the background is accounted for. In the case of the Cu 2p, the doublet sits on a shoulder of the O KLL Auger spectrum, which can be well described using a 6^th^-order polynomial function (see Fig. S.I. 2a†). For practical reasons, however, for the evaluation of our data, two linear functions (see Fig. S.I. 2b and related discussion for more details†) are used to account for the O KLL related background. In addition to this background model, the background of the Cu 2p spectrum itself is accounted for by an “active” Shirley-type background (see S.I., Python Script†). Finally, the spectra were fit with four Voigt profiles (*i.e.*, two doublets each representing the spin–orbit split 3/2 and 1/2 components of the 2p peaks), representing the main components ascribed to Cu(i) and Cu(ii) and six Voigt profiles for the complex satellite structure left to the main peaks, with two profiles each for the Cu(i) and Cu(ii) contribution to the 3/2 spin–orbit component. And one profile each for the Cu(i) and Cu(ii) contributions to the 1/2 spin–orbit component, as described in literature.^6,18^

For the Ni 2p, no background correction beyond the Shirley-type background is needed, but additional to the Ni 2p K_α_-satellites also the K_β_-satellite peaks of the Cu 2p spectra must be taken into account (further discussion about this in the following section).

To distinguish between Ni(ii) and Ni(iii), a different approach is needed. To separate the contributions to the Ni spectra, a different approach is needed. In this case it was assumed that only two contributions – Ni(ii) and Ni(iii) oxide – were present in the data set, and two sets of nine profiles each are used to represent each oxide respectively. There is some uncertainty in representing each oxidation state by a single spectrum in this way, since it neglects the possible contributions of Ni(ii) or Ni(iii) species with spectral shapes that differ from those of the oxides (*e.g.*, hydroxides). However, if such contributions are significant they will be reflected in the fit residues, and the fitting procedure can be modified through the introduction of the appropriate representative spectra either as an additional component or as a replacement of one of the two initial components. Four Voigt profiles (*i.e.*, two spin–orbit split doublets) are used for the main components and five Voigt profiles to represent the multiplet structure for one oxidation state of Ni.^19^ These fits show that the intensity of the Ni 2p_3/2_ to 2p_1/2_ peaks is not fixed to a ratio of 2 : 1 (as expected from their multiplicity), due to the impact of multiplet splitting.^20^ Examples of fits of Cu and Ni 2p photoemission spectra are shown in Fig. S.I. 1.† For quantification, the areas under the fitted 2p_3/2_ spectra, including corresponding satellites, are used 
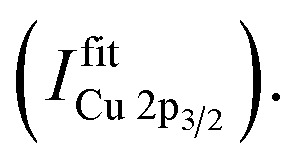
 To derive the composition, an expression like (eqn (1)) can be employed, replacing *I*^integration^_X_ by 
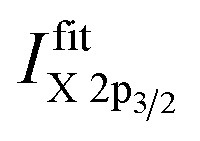
 using the cross section of the chosen core level (*σ*_3/2_ in Table S.I. 2†). A comparison of the results based on the *integration* and *fit* approaches is discussed in detail below to evaluate the possibility of using the former for fast quantification of large XPS data sets collected on material libraries.

## Results and discussion

**Fig. 3:**
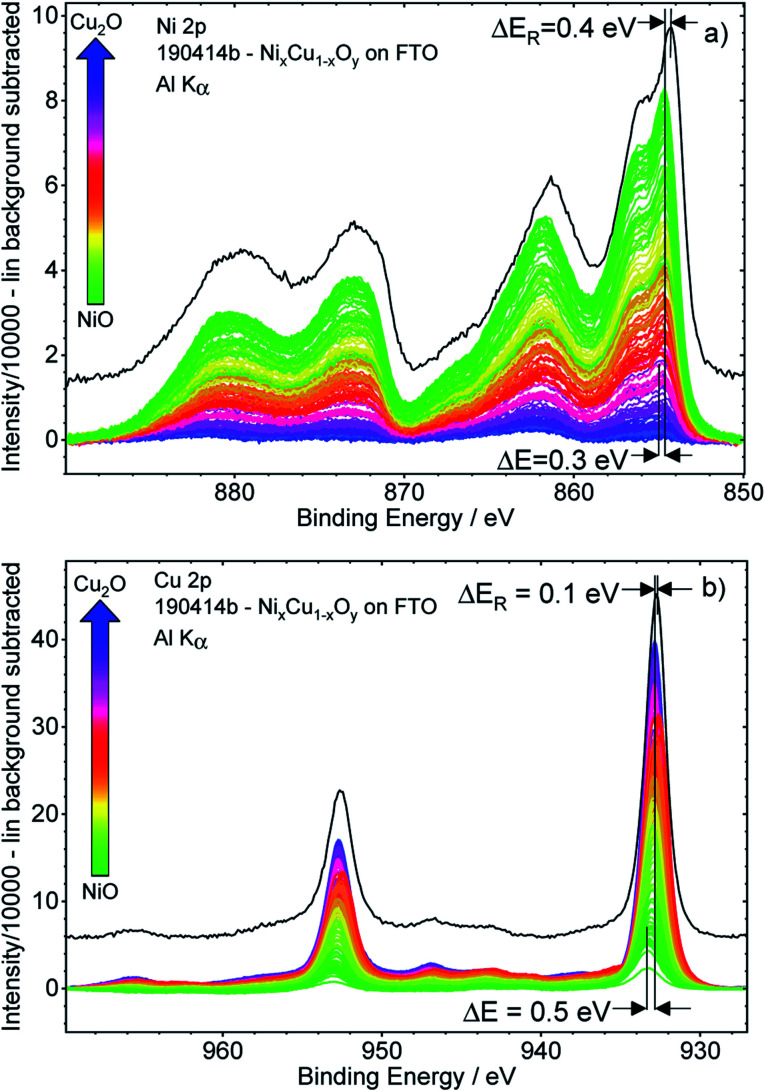
Ni 2p (panel a) and Cu 2p (panel b) XPS detail spectra after subtraction of a linear background of the 169 spots probed on the Cu_*x*_Ni_1−*x*_O_*y*_ library used for the integration approach. The given colour code indicates the nominal composition gradient: from NiO-rich (green) to Cu_2_O-rich (blue) with the BE shift Δ*E*. Reference spectra of NiO and Cu_2_O measured on a reference sample with Cu_2_O and NiO on FTO, respectively, are shown in black with the BE shift Δ*E*_R_.

Fig. 3 shows the background-subtracted Ni 2p (a) and Cu 2p (b) core level spectra of the 169 different measurement spots of the Cu_*x*_Ni_1−*x*_O_*y*_ library. It can be observed clearly that the Ni 2p and Cu 2p peak intensities follow the nominal NiO–Cu_2_O gradient (indicated by the color code) produced by the deposition process, as expected. The shape of the Ni 2p is strongly influenced by multiplet splitting. For high Ni contents, the (green) spectra agree well with the reference spectrum of a NiO sample (shown as a black line in Fig. 3a). However, the spectra are slightly shifted to a higher BE compared to the reference. For lower Ni contents, the shapes of the Ni 2p spectra start to deviate significantly from that of NiO, with the difference becoming increasingly pronounced for decreasing Ni contents (see normalized Ni 2p spectra in Fig. S.I. 3a†). However, close inspection of the spectra reveals that the spectral change is mainly caused by an increasing contribution of an Al K_β_ excitation satellite of the Cu 2p spectra (see detailed discussion in conjunction with Fig. S.I. 3b and c†) with decreasing Ni, (*i.e.*, increasing Cu) content. After correcting this additional background effect, we find a (mainly) unvarying spectral shape of the Ni 2p spectra.

The peak positions of the (blue) Cu 2p spectra in Fig. 3b, representing Cu_2_O-rich regions, show an absence of pronounced Cu(ii)-related satellite features at 940–945 eV.^18^ Comparison to the Cu_2_O reference spectrum (in black, at slightly lower BE) also confirms that Cu is mainly in the +1 oxidation state (*i.e.*, Cu(i)), as expected considering the use of Cu_2_O as precursor material in the PLD process. However, close inspection of the data reveals that with increasing Ni content, a broadening of the 2p_3/2_ peak at ∼933 eV and a relative increase of the Cu(ii)-related satellite intensity occurs (see normalized Cu 2p spectra in Fig. S.I. 4 and example fits Fig. S.I. 1†). This result indicates a change in the chemical composition and oxidation state of Cu – particularly in the NiO-rich regime, as also supported by the fits shown in Fig. S.I. 1.† However, the decrease of the Cu(i)/Cu(ii) ratio is not related to the increase in Cu(ii), but rather to the decreasing overall Cu content towards the NiO-rich region. Note that great care has been taken to minimize air exposure of the Cu_*x*_Ni_1−*x*_O_*y*_ library after deposition.

The Ni 2p and Cu 2p spectra measured on individual library spots show a BE shift depending on the composition. Moving from the Cu_2_O-rich to NiO-rich area, the Ni 2p_3/2_ BE decreases from 855.0 eV to 854.7 eV (≈−0.3 eV) and the Cu 2p_3/2_ BE increases from 932.8 eV to 933.3 eV (≈+0.5 eV).

**Fig. 4:**
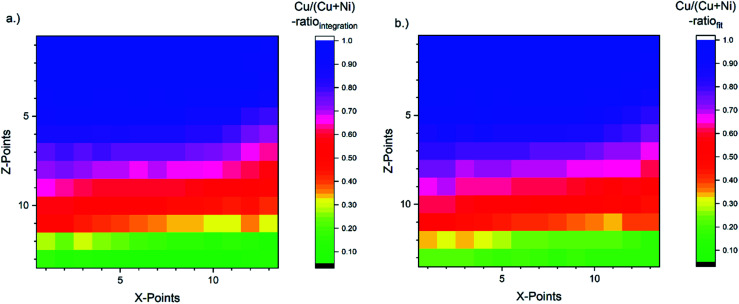
[Cu]/([Cu] + [Ni]) ratio for all probed 169 spots (depicted by means of a 13 × 13 grid) of the Cu_*x*_Ni_1−*x*_O_*y*_ combinatorial material library derived by using eqn (1). The color-coded map (a) is obtained by using the peak areas derived by the linear background subtraction and integration and the composition depicted in map (b) is based on the peak areas derived by fitting the XPS spectra. The [Cu]/([Cu] + [Ni]) ratio indicates a strong [Cu]/([Cu] + [Ni]) gradient along the *Z*-axis as expected. It ranges from a [Cu]/([Cu] + [Ni]) ratio of around 1.0 (1.0) ± 0.01 in the Cu_2_O-rich region to 0.05 (0.15) ± 0.01 in the NiO-rich region for the integration (fit) approach.

The corresponding [Cu]/([Cu] + [Ni]) ratio for all 169 probed spots, as derived with the fast *integration* and detailed *fit* approaches, using eqn (1), are shown in Fig. 4a and b, respectively, by means of color-coded 13 × 13 maps. The derived [Cu]/([Cu] + [Ni]) ratio according to the *integration* approach ranges from ≈0.05 to 1.00 (±0.01) when going from the NiO-rich area of the library sample to the Cu_2_O-rich regime. As shown in Fig. 3a and S.I. 3a,† the Ni 2p and the Cu 2p signal never completely vanish even in the most Cu_2_O-rich or NiO-rich regions, respectively. While this leads to a quantifiable amount of Cu in the most NiO-rich region, the small amount of Ni is not sufficient to have a significant impact (beyond the experimental uncertainty) on the derived [Cu]/([Cu] + [Ni]) ratio in the most Cu_2_O-rich region. This imbalance is also clearly seen in the [Cu]/([Cu] + [Ni]) maps in Fig. 4 and can be explained by NiO and Cu_2_O having different ablation rates. In the PLD deposition process, this results in different amounts of NiO and Cu_2_O being deposited despite using an equal number of pulses with the same laser energy.

**Fig. 5:**
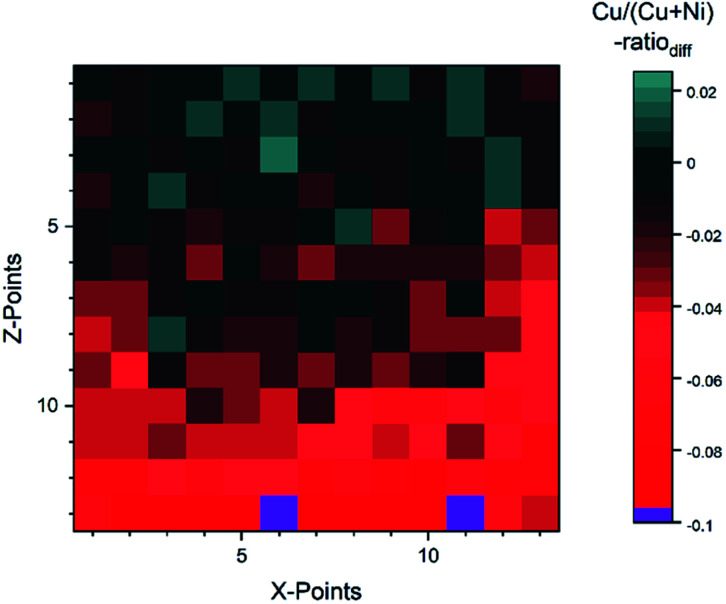
Absolute difference between the Cu/(Cu + Ni)-ratio derived by the integration and fit approaches. The two purple spots (162 and 167) are outliers with a difference ≥10%.

For the *fit* approach, [Cu]/([Cu] + [Ni]) ranges from ≈0.15 to 1.00 (±0.01) and shows similar behavior in the *Z*-direction as derived by the integration approach. To evaluate the differences between the two quantification approaches, the absolute [Cu]/([Cu] + [Ni]) ratio difference (=[Cu]/([Cu] + [Ni])_integration_ − [Cu]/([Cu] + [Ni])_fit_) is computed and shown by means of a color-coded map in Fig. 5 (for completeness, the quantified [Cu]/([Cu] + [Ni])) values for the 13 × 13 grids for the different quantification approaches and the computed deviation, are shown in (Fig. S.I. 5–7†). We find the deviation between the [Cu]/([Cu] + [Ni]) ratios derived by the two quantification approaches to be under ±0.10 except for two points (spot 162: −0.12 and spot 167: −0.10, purple spots in Fig. 5), with a range of −0.09 to +0.02.

This result is remarkable considering the different quantification approaches and how backgrounds are considered (linear *vs.* Shirley background for Ni 2p and linear *vs.* Shirley + polynomial background for Cu 2p), with the polynomial likely being the biggest source of uncertainty. Spots 1–52 (rows 1–4) have a small deviation of under 0.03. And even for spots 54–142 (rows 5–11) the deviation is ≤ |−0.05|, while in the last two rows (spots 144–169) the *integration* approach significantly underestimates the presumably more accurate *fit*-derived [Cu]/([Cu] + [Ni]) ratio ([Cu]/([Cu] + [Ni]) ratio_diff_ = −0.05… − 0.12). The underestimation in the most NiO-rich region is due to the background choice for the Cu 2p spectra. To automate the fitting, the most Cu_2_O-rich region (spots 1–13) was used to define the start/end point of the linear background fitting. But with the smaller Cu 2p intensity, the contribution of the right shoulder of the O KLL to the signal, which overlaps with the Cu 2p_1/2_ (see Fig. S.I. 8b†) increases. This leads to a “negative intensity”, which artificially decreases the integral-derived area. The problem can be prevented by choosing a suitable background for each spectrum; however, this intervention violates the desired “hands-off” approach for fast quantification. Another approach would be to consider rows 14 and 15 as a ‘separate’ region of interest employing an optimized background correction for this Cu_2_O-poor region. Alternatively, one could exploit statistical methods for data evaluation. Using *e.g.*, Grey relational analysis, we could show that spectra deconvolution is less affected by background effects.^21^

A thorough *fit* analysis of the data can reveal additional chemical structure information, *e.g.*, different species and oxidation states and it also allows to more flexibly consider changing complicated background contributions – like the O KLL related background in the case of Cu 2p. The *integration* approach, then again, is expected to be robust and significantly faster and thus assumed to be more relevant to efficiently evaluate large data sets, as expected to be generated for even more complex combinatorial material libraries or during *operando*/*in situ* experiments where fast feedback can also be used in experiment control.

These results on data evaluation schemes can be optimized to show the potential of XPS also in combinatorial materials research. However, for XPS to become a valid high-throughput analysis tool, data acquisition has to be significantly accelerated. In the current case, the measurement time alone amounted to 200 hours per core level in total. A straightforward way to reduce measurement time is to use a more brilliant light source than the laboratory-based twin anode X-ray source that was used here. Using, *e.g.*, the soft X-ray branch of the two-colour beamline of EMIL^22^ instead would increase the overall X-ray photon flux by a factor of 30. Considering the focused beam spot (of approx. 30 μm × 25 μm), the photon flux *density* would be enhanced by almost 5 orders of magnitude (see S.I.: Photon flux†). However, note that with high-flux densities, beam-induced artifacts (*i.e.*, beam damage) might become an issue for irradiation-sensitive samples. In any case, it seems feasible to significantly reduce the measurement time to a few (or even below) 1 hour for the 169 spot library as measured here. In addition, the *integration* quantification approach does not require collecting data with high energy resolution, so a fast sweep with high pass energy or even a survey spectrum could be enough to get most of the compositional information. Fully exploiting the focused beam spot would then allow to increase the number of probed spots, if fast changing sample properties should require this.

Automated spectra processing schemes have to be developed to decrease data evaluation times that allow for ‘real-time’ data processing and evaluation. Using the *integrated* approach can (only) be a start. This may enable ‘on-the-fly’ analysis, where, during the measurement, spots of interest are automatically preselected and further investigated. The detailed *fit* analysis can then be done for data of selected spots of interests, further reducing data acquisition time.

## Conclusions

We have presented XPS data collected for a large-scale (72 × 72 mm^2^) Cu_*x*_Ni_1−*x*_O_*y*_ combinatorial material library. The measurements were performed to evaluate to what extent XPS can be optimized to become a valid high-throughput method for obtaining relevant material properties of such combinatorial libraries. For that, 169 spots on the large-scale sample were characterized by using the moderate spatial resolution of a state-of-the-art commercially available electron analyzer. Two different quantification approaches were presented. A relatively coarse, but fast and robust approach based on peak area determination by *integration* and a more detailed, but more resource-consuming approach based on area determination by peak *fit*. Both procedures reveal a clear [Cu]/([Cu] + [Ni]) composition gradient along the sample. The deviation between the composition derived by the *integration* and *fit* approach is (except for two points out of 169) under 10% absolute, suggesting that the former can be used in fast quasi ‘real-time’ data evaluation paving the way for ‘on-the-fly’ analysis and automated measurement spot selection in the future. Together with the fact that it seems feasible to significantly reduce measurement time by using more brilliant light sources, the road for XPS to become a real high-throughput analysis tool for combinatorial materials research is now wide open.

## Conflicts of interest

There are no conflicts to declare.

## Supplementary Material

RA-012-D1RA09208A-s001
