# Supplementary material for: Prospect of making XPS a high-throughput analytical method illustrated for a CuxNi1−xOy combinatorial material library
Source: RSC Adv. 2022 Mar 11;12(13):7996–8002. doi: 10.1039/d1ra09208a (PMC8982450; doi:10.1039/d1ra09208a)
Supplement: RA-012-D1RA09208A-s001 [file RA-012-D1RA09208A-s001.pdf]

Electronic Supplementary Material (ESI) for RSC Advances.
This journal is © The Royal Society of Chemistry 2022

Supporting Information

Prospect of making XPS a high-throughput analytical method illustrated for a CuxNi1-xOy combinatorial material library

L. C.W. Bodenstein-Dresler[1], A. Kama[2], J. Frisch[1], C. Hartmann[1], A. Itzhak[2], R.G. Wilks[1,3], D. Cahen[2,4], M. Bär[1,3,5,6]

[1]Dept. Interface Design, Helmholtz-Zentrum Berlin für Materialien und Energie GmbH (HZB), Berlin, Germany

[2] Department of Nanotechnology and Advanced Materials, Bar-Ilan University (BIU), Ramat Gan, Israel

[3]Energy Materials In-Situ Laboratory Berlin (EMIL), HZB, Berlin, Germany

[4]Department of Materials & Interfaces, Weizmann Institute of Science (WIS), Rehovot, Israel

[5]Department of Chemistry and Pharmacy, Friedrich-Alexander-Universität Erlangen-Nürnberg (FAU), Erlangen, Germany

[6]Helmholtz-Institute Erlangen-Nürnberg for Renewable Energy (HI ERN), Berlin, Germany

Calculation of possible elemental compositions

The combination of only three elements and composition step changes of 10 % for each element results in over 5000 possible compositions is based on the following consideration: If we assume three elements (e.g., Ni, Cu, O) and composition step changes of 10%, this results in 11 different amounts for a specific element (0, 0.1, 0.2 … 1). Thus, the total amount of possible combinations can then be calculated according to:

$$\binom{33}{3} = \frac{33!}{(33-3)!} * 3 = 5456$$

Exemplary data and fits

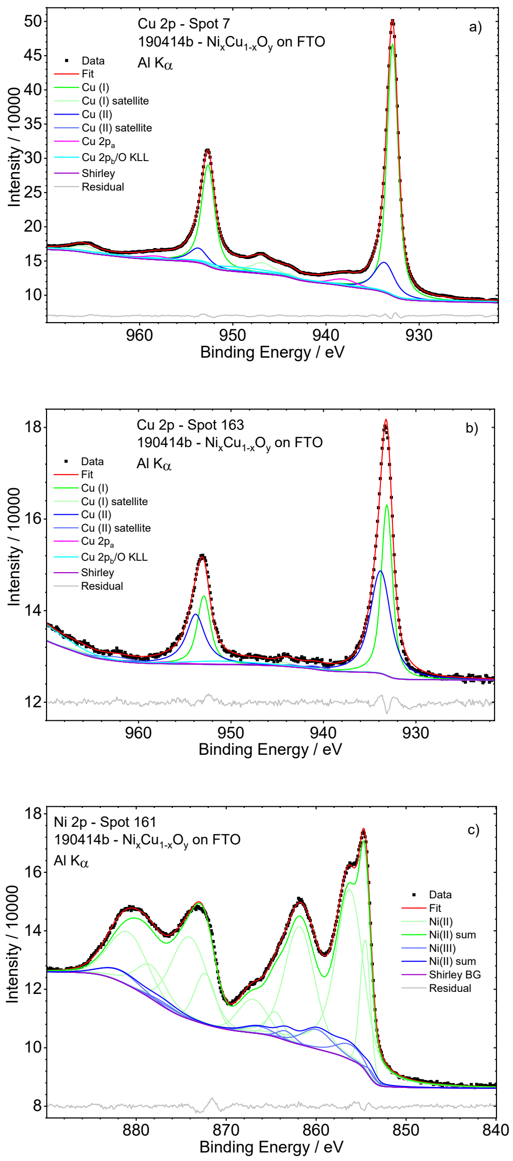

Figure S.I. 1: Exemplary data and fits of the $Cu_xNi_{1-x}O_y$ sample library. In panel a & b), the Cu 2p spectra of spot 7 and spot 163 are shown together with the respective fits: Shirley background (BG), Voigt profiles for Cu(I), Cu(II) oxidation state contributions including respective satellite features as well as Cu $2p_a$ (loss feature) and Cu $2p_b$ (highly complex O KLL related BG artefact). In panel c) an exemplary fit of the Ni 2p spectrum collected for spot 161 is depicted, revealing the contribution of Ni(II) and Ni(III) oxidation states. The Ni(II) shape was taken from a NiO reference sample (see Fig. 3a in the main manuscript). The difference between the reference and the measured spectra is assigned to Ni(III). Below each spectrum the residual, i.e. the difference between experimental data and fit is shown.

Spot and sample position

The spots in region 1 (column 3-11 and row 7-13, spots 81-167) were measured in the range of X= [-22, 18] mm, Z= [209.5, 239.5] mm and R_0= 0°. Region 3 (same column, row 1-6, (spots 3-76)) was measured with R_0=180°. Region 2 (column 1-2 at row 3-11, spots 27-132) was measured with X= [-22, -18] mm, Z= [209.5, 214.5] mm and rotated by 90°, while the one on the opposite side – region 4 - (column 12-13, spots 38-143) was rotated by R_0=270°. The corner spots, regions 5-8, were all measured with X= [-5.53, -2, 1.53] mm, Z= [197.57, 200.9, 204.5] mm and rotated by R_0= [45, 135, 225, 315]° respectively (**Table S.I. 1**).

Table S.I. 1: Manipulator coordinates of the probed 169 different sample spots (see Figure 2 in main manuscript) addressed by eight different measurement regions of constant rotation.

Region	Column	Row	Spots	X /mm	Z /mm	R_0 /°
1	3-11	7-13	81-167	[-22 ; 18]	[209.5 ; 239.5]	0
2	1-2	3-11	27-132	[-22 ; 18]	[209.5 ; 214.5]	90
3	3-11	1-6	3-76	[-22 ; 18]	[209.5 ; 239.5]	180
4	12-13	3-11	38-143	[-22 ; 18]	[209.5 ; 214.5]	270
5	1-2	12-13	144-158	[-5.53, -2, 1.53]	[197.57, 200.9, 204.5]	45
6	1-2	1-2	1-15	[-5.53, -2, 1.53]	[197.57, 200.9, 204.5]	135
7	12-13	1-2	12-26	[-5.53, -2, 1.53]	[197.57, 200.9, 204.5]	225
8	12-13	12-13	155-169	[-5.53, -2, 1.53]	[197.57, 200.9, 204.5]	315

IMFP and composition determination

Since the IMFP changes depending on the elemental composition an IMFP of the element *Y* (Cu or Ni) based on elemental composition *X* was calculated via

$$IMFP_X^Y = X * IMFP^{IMFP(553.7eV)/Å,\,Y} + X * IMFP^{IMFP(631.5eV)/Å,Y} \quad \text{(Eq. S.I. 1)}$$

E.g. for $X = \frac{Cu}{Cu + Ni} = 0.75$:

$$IMFP_{0.75}^{Cu} = 0.75 * IMFP^{IMFP(553.7eV)/Å,\,Cu} + 0.25 * IMFP^{IMFP(631.5eV)/Å,Cu}$$

$$= 11.75\ Å$$

$$IMFP_{0.75}^{Ni} = 0.75 * IMFP^{IMFP(553.7eV)/Å,Ni} + 0.25 * IMFP^{IMFP(631.5eV)/Å,Ni}$$

$$= 12.94\ Å$$

with $IMFP^{IMFP(553.7eV)/Å,Cu}$ = 11.98 Å, $IMFP^{IMFP(631.5eV)/Å,Cu}$ = 13.18 Å, $IMFP^{IMFP(533.7eV)/Å,Ni}$ = 11.09 Å, $IMFP^{IMFP(631.5eV)\,/Å,Ni}$ = 12.23 Å, taken from the QUASES software [QUA19] and depicted in **Table S.I. 2**.

This IMFP was then inserted in **Eq. 1** given in the main text and the new ratio is calculated. This was repeated in a loop of 5000 iterations, which takes the elemental composition value of the previous iteration and then uses the corresponding new IMFP value.

Table S.I. 2: In this table the inelastic mean free path λ for Cu_2O, CuO, and NiO are shown (taken from [QUA19]). For the IMFP calculation in $Cu_XNi_{1-x}O_y$, the IMFP of Cu_2O was chosen to represent the copper oxide contribution, since it is the main Cu-related species.

	Cu_2O	CuO	NiO
$\lambda_{533.7eV}$ / Å	11.98	11.90	11.09
$\lambda_{631.5eV}$ / Å	13.18	13.12	12.23

Transmission function

The transmission function (TF) is obtained by determining a mathematical function in dependence of the kinetic energy, which scales a measured survey spectrum to a TF-corrected survey. For the Argus analyzer it seems to be a linear function for KE > 350 eV (which is the case for our Ni 2p and Cu 2p). For the sake of simplicity for the fast analysis we used a value in the middle of our spectra´s energy range. Resulting in the factor shown in **Table S.I. 3.**

Table S.I. 3: Values for the element-specific photoionization cross section σ (taken from [Trz06]) and the analyzer specific transmission function (TF). For the σ_{tot} the sum of $\sigma_{3/2}$ and $\sigma_{1/2}$ is used.

	Cu 2p	Ni 2p
$\sigma_{1/2}$	8.8	9.4
$\sigma_{3/2}$	17.47	19.35
σ_{tot}	26.27	28.74
TF	0.724	0.730

Cu 2p: Background considerations

The Cu 2p spectrum is overlapped with an O KLL related background feature. A 6th order polynomic function was chosen to simulate the right shoulder of the O KLL peak. But since the Cu 2p energy range only reaches from 915 – 970 eV, it is not possible to calculate the right polynomial for each spectra, since each one has a different Cu 2p peak/O KLL background intensity ratio itself. To mitigate this issue, two linear functions are used as an approximation. One is the average gradient from 915 – 921 eV and the other one uses a group of points around 920 eV and 926 eV. Then an average gradient is used and extracted up to 970 eV. This is done for all following Cu 2p spectra

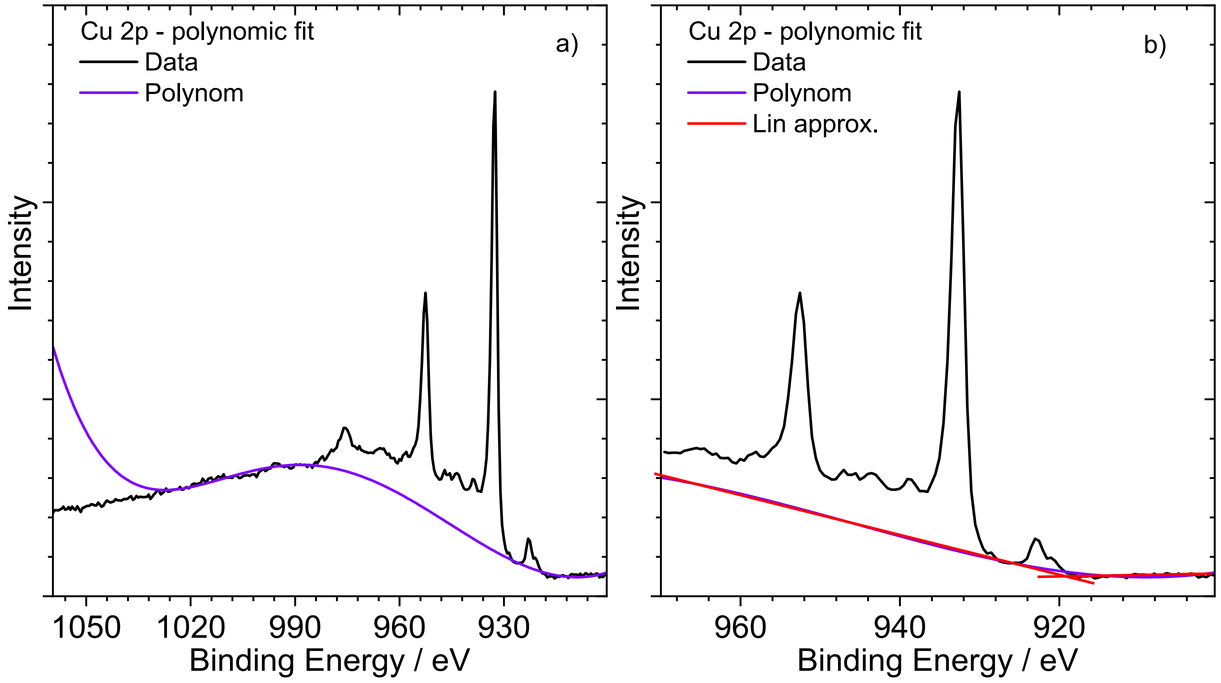

Figure S.I. 2: Region of the Cu 2p line with O KLL related background feature. Exemplary 6th order polynomic fit ('Polynom') of the background is shown in a). In b) a magnified view of the same Cu 2p spectrum with an approximation of the polynomic background fit by two linear functions ('Lin approx') ranging from 915-921 & 921-927 eV is shown.

Spectral changes of Ni 2p and Cu 2p

In order to emphasize the spectral changes of the Cu 2p and Ni 2p spectra, they are shown in Figure S.I. 3 and 4 normalized to [0;1]. Note that the seeming intensity cut-off is caused by the low-signal-to-noise ratio in for the Cu_2O-rich measurement sports and the normalization procedure. The comparison with reference spectra reveals that the spectral intensity around the shake-up peak at 863 eV in the Ni 2p spectra can also partly be attributed to K_β-line excited Cu 2p spectra. This is especially visible for the Cu_2O-rich measurement spots, where the shake-up peak is higher than the Ni $2p_{3/2}$. The results of subtracting the K_β-line excited spectral intensity from the experimental data is shown for measurement spots 16 and 85 in Figure S.I. 3 b) & c).

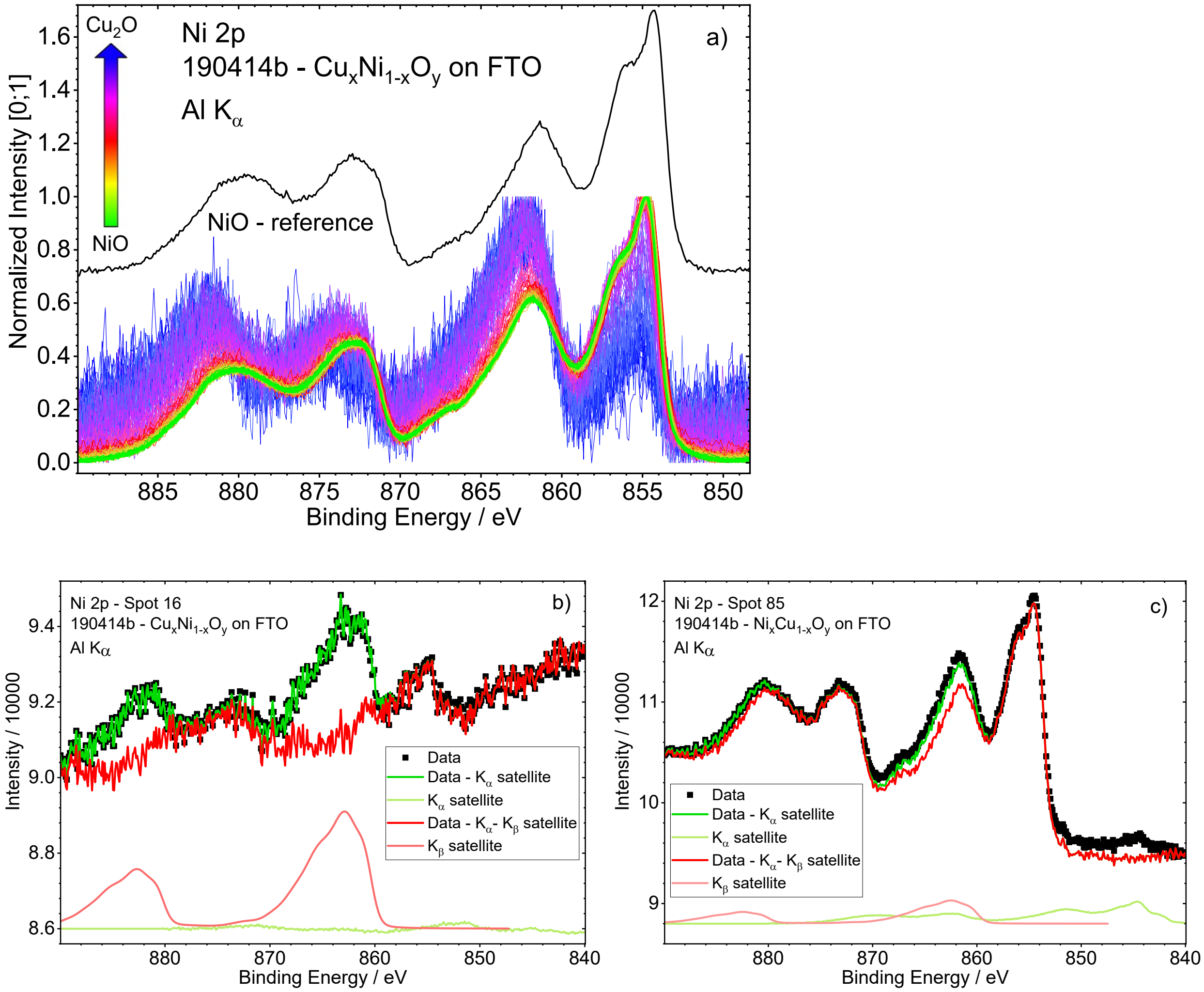

Figure S.I. 3: Panel a) shows the normalized Ni 2p spectra of the $Cu_xNi_{1-x}O_y$ sample library together with a NiO (99.9% pure, Kurt J. Lesker Company) reference spectrum (black). Panel b) & c) show two Ni 2p example spectra of measurement spots 16 and 85 with the K_α and K_β excited Cu 2p related spectral intensities subtracted.

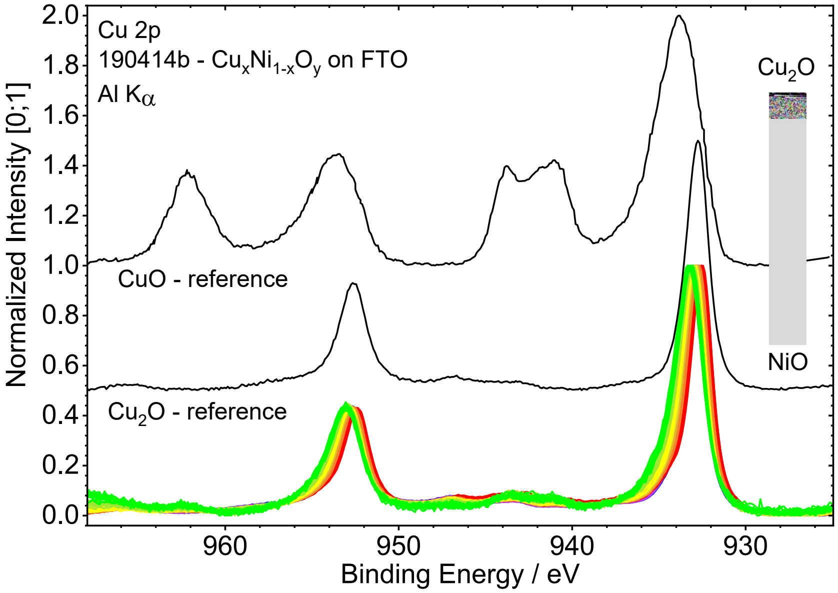

Figure S.I. 4: Normalized Cu 2p spectra of the $Cu_xNi_{1-x}O_y$ sample library together with two reference spectra (Cu_2O and CuO (99.9% pure, Kurt J. Lesker Company), black)).

Confidence intervals and [Cu]/([Cu]+[Ni]) ratios

In order to estimate the spread between min. to max. ratio in the MO, it is necessary to calculate the minimum and maximum content of both Cu and Ni in the sample according to the error margins of the measurement instrument and given theoretical values.

But we have no information about the probability distribution and per value only a relative error. Due to this, one expects a symmetric (Gaussian) distribution. But for the Gaussian distribution sigma values are necessary which are also not given. Because of all these reasons, the distribution must be approximated as a symmetric function with upper and lower limit.

For this the weighting of the possible minimal error value for e.g. Cu (ΔCu_{min}) is calculated by determining the ratio between the minimal expected value (n_{min}) and the expected value/measured Intensity (I_{Cu}) (**Eq. S.I. 2a**). To get the minimal we make the numerator as small as possible and the denominator as big as possible. Therefore we use the minimum relative value "$1 - error$" in the numerator and the maximum relative values "$1 + error$" in the denominator (**Eq. S.I. 2b**). In case of the n_{max} procedure it`s vice versa.

$$\Delta Cu_{min} = \frac{n_{min}}{I_{Cu}} \quad \text{(Eq. S.I. 2a)}$$

$$= \frac{1 - error_{Intensity}^{integration, fit}}{(1 + error_{\sigma}) * (1 + error_{\lambda}) * (1 + error_{TF})} \quad \text{(Eq. S.I. 2b)}$$

For the photoionization cross section values from **Tab S.I. 3** an estimation of $error_{\sigma}$ = 20% was used. To the IMFP values from **Tab S.I. 2** an $error_{\lambda}$= 10% was assigned and $error_{TF}$ = 5% for the transmission function values from **Tab S.I. 3**. The $error_{Intensity}$ is the estimated error of the fit, which was chosen for each spot individually by looking at the spectra and overestimate the possible error value. The $error_{Intensity}$ for the *integration* was estimated by deciding the biggest possible error of area calculation due to the linear BG approximation for each spectra individually. For the Cu spectra it was mainly 5%, but in the last 3 rows the error was up to 15% (Spots 131-143), 40% (spots 144-156) and for the last row 50% (spots 157-169) estimated. The reason for this big error is exemplary shown in **Fig S.I. 8b**. The Ni value error is manly <5%, except for the first 4 rows (50% for spot 1-26 and 30-40% for spots 27-52) which originates from the low intensity and therefore possible big fitting error. The $error_{Intensity}$ for the *fit* also was estimated by deciding the biggest possible error for each fit individually and was for the Cu fits to 5%, while for the Ni fits from 5-50%. This big value (50% for spot 1-26 and 30% for spots 27-39) follows the same reasoning as stated above.

With these the absolute upper and lower error value estimation of the [Cu]/([Cu]+[Ni]) ratio $\Delta_{min, max}^{integration, fit}$ get`s derived each for the *integration* and *fit* approach (**Eq. S.I. 2c**). To calculate e.g. the min

value for the ratio the weighting of the error value $\Delta Cu_{min}, \Delta Ni_{min}$ gets multiplied by the intensity of the corresponding element $I_{Cu,\, Ni}$.

$$\Delta_{min,\, max}^{integration,\, fit} = \frac{I_{Cu} * \ \Delta Cu_{min,max}}{I_{Cu} * \ \Delta Cu_{min.max} + I_{Ni} * \ \Delta Ni_{max,min}} \qquad \text{(Eq. S.I. 2c)}$$

1.00	0.98	1.00	0.99	1.00	1.00	1.00	0.99	1.00	0.98	1.00	0.97	0.95	+0.01<br>-0.01
0.98	0.98	0.99	1.00	0.98	1.00	0.97	0.99	0.99	0.99	0.99	0.96	0.94	+0.01<br>-0.02
0.99	0.98	0.98	0.99	0.98	1.00	0.98	0.97	0.97	0.98	0.97	0.97	0.92	+0.01<br>-0.02
0.95	0.97	0.97	0.96	0.95	0.97	0.95	0.97	0.96	0.96	0.95	0.94	0.89	+0.01<br>-0.02
0.92	0.94	0.93	0.93	0.93	0.93	0.94	0.94	0.90	0.90	0.89	0.82	0.79	+0.01<br>-0.02
0.87	0.87	0.87	0.86	0.88	0.86	0.85	0.85	0.84	0.83	0.80	0.76	0.71	+0.01<br>-0.02
0.78	0.79	0.78	0.79	0.78	0.78	0.78	0.77	0.76	0.73	0.72	0.68	0.63	+0.02<br>-0.02
0.71	0.73	0.71	0.70	0.69	0.68	0.69	0.68	0.67	0.65	0.63	0.62	0.57	+0.02<br>-0.02
0.65	0.64	0.62	0.60	0.60	0.60	0.59	0.59	0.57	0.55	0.55	0.53	0.49	+0.02<br>-0.03
0.58	0.58	0.53	0.53	0.52	0.51	0.51	0.48	0.47	0.45	0.44	0.45	0.41	+0.03<br>-0.04
0.50	0.50	0.44	0.41	0.39	0.38	0.36	0.34	0.33	0.31	0.31	0.35	0.32	+0.06<br>-0.04
0.27	0.24	0.29	0.25	0.23	0.19	0.17	0.16	0.15	0.13	0.15	0.13	0.09	+0.11<br>-0.05
0.16	0.12	0.14	0.11	0.09	0.07	0.08	0.08	0.08	0.07	0.05	0.09	0.11	+0.07<br>-0.05

*Figure S.I. 5: [Cu]/([Cu]+[Ni]) ratio obtained by the integration approach. With an estimation of the Cu content under consideration of the measurement dependent confidence intervals $\Delta_{min,\, max}^{integration}$ on the right (after **S.I. Eq. 2a**).*

1.00	0.99	1.00	0.99	0.99	1.00	0.99	0.99	0.99	0.99	0.99	0.98	0.97	+0.01<br>-0.03
1.00	0.99	0.99	0.99	0.98	0.99	0.98	0.99	0.99	0.99	0.98	0.97	0.95	+0.01<br>-0.03
0.99	0.98	0.99	0.99	0.99	0.98	0.98	0.98	0.98	0.98	0.98	0.96	0.93	+0.01<br>-0.03
0.97	0.97	0.96	0.97	0.95	0.97	0.97	0.97	0.97	0.96	0.95	0.93	0.90	+0.01<br>-0.02
0.93	0.94	0.94	0.95	0.94	0.94	0.94	0.93	0.93	0.91	0.89	0.86	0.82	+0.02<br>-0.02
0.88	0.89	0.88	0.89	0.88	0.88	0.88	0.87	0.86	0.85	0.82	0.79	0.75	+0.02<br>-0.02
0.81	0.82	0.79	0.79	0.79	0.79	0.78	0.77	0.77	0.76	0.72	0.72	0.68	+0.03<br>-0.04
0.75	0.76	0.70	0.71	0.71	0.70	0.69	0.70	0.68	0.68	0.66	0.65	0.62	+0.04<br>-0.04
0.68	0.69	0.63	0.63	0.63	0.62	0.62	0.61	0.60	0.57	0.56	0.58	0.54	+0.04<br>-0.04
0.62	0.62	0.57	0.55	0.55	0.55	0.53	0.53	0.53	0.51	0.49	0.51	0.46	+0.04<br>-0.04
0.54	0.54	0.47	0.45	0.43	0.42	0.41	0.39	0.37	0.36	0.34	0.40	0.39	+0.03<br>-0.03
0.34	0.31	0.34	0.31	0.28	0.24	0.24	0.22	0.22	0.21	0.21	0.20	0.16	+0.02<br>-0.02
0.22	0.21	0.22	0.19	0.17	0.19	0.17	0.16	0.17	0.15	0.15	0.15	0.15	+0.01<br>-0.01

*Figure S.I. 6: [Cu]/([Cu]+[Ni]) content obtained by the fit approach. With an estimation of the Cu content under consideration of the measurement dependent confidence intervals $\Delta_{min, max}^{fit}$ on the right (after **S.I. Eq. 2a**).*

0.00	-0.01	0.00	0.00	0.01	0.00	0.01	0.00	0.01	-0.01	0.01	-0.01	-0.02
-0.02	-0.01	0.00	0.01	0.00	0.01	-0.01	0.00	0.00	0.00	0.01	-0.01	-0.01
0.00	0.00	-0.01	0.00	-0.01	0.02	0.00	-0.01	-0.01	0.00	-0.01	0.01	-0.01
-0.02	0.00	0.01	-0.01	0.00	0.00	-0.02	0.00	-0.01	0.00	0.00	0.01	-0.01
-0.01	0.00	-0.01	-0.02	-0.01	-0.01	0.00	0.01	-0.03	-0.01	0.00	-0.04	-0.03
-0.01	-0.02	-0.01	-0.03	0.00	-0.02	-0.03	-0.02	-0.02	-0.02	-0.02	-0.03	-0.04
-0.03	-0.03	-0.01	0.00	-0.01	-0.01	0.00	0.00	-0.01	-0.03	0.00	-0.04	-0.05
-0.04	-0.03	0.01	-0.01	-0.02	-0.02	0.00	-0.02	-0.01	-0.03	-0.03	-0.03	-0.05
-0.03	-0.05	-0.01	-0.03	-0.03	-0.02	-0.03	-0.02	-0.03	-0.02	-0.01	-0.05	-0.05
-0.04	-0.04	-0.04	-0.02	-0.03	-0.04	-0.02	-0.05	-0.06	-0.06	-0.05	-0.06	-0.05
-0.04	-0.04	-0.03	-0.04	-0.04	-0.04	-0.05	-0.05	-0.04	-0.05	-0.03	-0.05	-0.07
-0.07	-0.07	-0.05	-0.06	-0.05	-0.05	-0.07	-0.06	-0.07	-0.08	-0.06	-0.07	-0.07
-0.06	-0.09	-0.08	-0.08	-0.08	-0.12	-0.09	-0.08	-0.09	-0.08	-0.10	-0.06	-0.04

Figure S.I. 7: Absolute deviation of the [Cu]/([Cu]+[Ni]) ratio: “integration approach” minus “fit approach”

Consideration of background (BG) for the *integration* approach

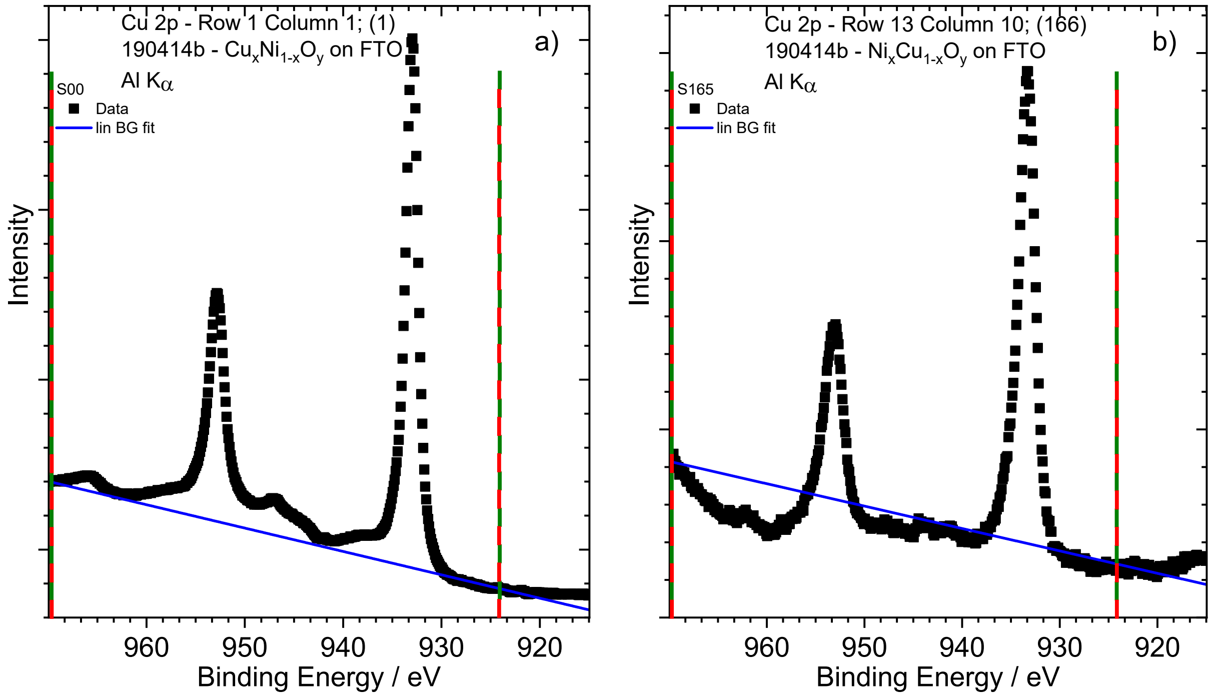

Figure S.I. 8: Linear BG fit of the Cu 2p region with selected fit boundaries (924.05 - 969.85 eV) in green and integration range (same) in red. The underestimation of Cu 2p in panel b) is clearly visible, which leads to the increased error in the area calculation for low Cu contents.

Photon flux at EMIL

The values listed below are the photon flux numbers at the soft x-ray branch (UE 48) of the two-color EMIL beamline [HZB21] for open front-end aperture settings; 100 microns exit slit size and a c_{ff} of 2.25 at a BESSY II ring current of 300 mA. These are the standard beamline settings that were also used in the RAY tracing calculation for beamline design.

For these conditions, the total photon flux varies in between $1.00x10^{13}$ photons/s at photon energies of about 100 eV to a maximum of $1.37x10^{13}$ photons/s at 600 eV and then decreases according to the lower PGM transmission at higher energies providing 8.4 $x10^{11}$ photons/s at 1487 eV.

For a 588 μm^2 focus ellipse shaped (30 µm x 25 µm HxV), this results in total photon flux density values of:

1.7 $x10^{18}$ photons/s/cm^2 @100 eV

2.32 $x10^{18}$ photons/s/cm^2 @600 eV

1.42 $x10^{17}$ photons/s/cm^2 @1487 eV

In comparison, the photon flux density of the lab source used in the described XPS measurements is $5x10^{12}$ photons/s/cm^2 [SPE18] at Al K_α 1486.58 eV, at this energy the EMIL soft x-ray beamline delivers 1.42 $x10^{17}$ photons/s/cm^2, i.e. almost 5 orders of magnitude more.

Python script

The Python program was written in version 3.8 and had several tasks to fulfill.

- It should be able to fit different shaped peaks (e.g. Voigt, Gaussian)
- The Shirley background (SBG) should be coupled to the each peak individually
- The SBG should change with the change of the area/width/height of the assigned peak (active Shirley)
- Multiple spectra should be fitted at the "same" time (same corresponding peaks should be linked)

Under an "active" Shirley BG we understand a BG which changes its shape according to its assigned peak exemplary shown in the *CasaXPS* manual [Cas06]. Many programs, like *fityk* [fit20] calculate a static BG by using the area of the complete spectra and do not take two things into account.

The first is when using the formula to calculate the Shirley BG curve

$$S(E) = I_2 + \kappa \frac{A_2(E)}{A_1(E) + A_2(E)} = I_2 + \kappa \frac{A_2(E)}{A_{total}(E)} \qquad \text{(S.I. Eq. 3)}$$

with I_2 as the lower starting point, κ the intensity step, A_1 as the area of the peak left and A_2 as the area already scanned through, the total area A_1+A_2 is a lot greater if only a single Shirley function for all peaks is used instead of a single one for each peak separately.

The second part is with the changing BG, the peak heights & shapes may differ during the fitting process as well which would then change the Shirley and so on until the changes get negligible during fitting. Especially when multiple peaks are overlapping the Shirley BG will have a different shape and might change the peak area on top of it.

This method was used and transcripted into Python. The main package used was the *lmfit* package [New14] using the Levenberg-Marquardt algorithm [Lev44, Mar63]. This provides a lot of pre-set functions like Gaussian, Lorentzian, Voigt or Doniach type profiles. To these a Shirley function, based on **S.I. Eq. 3**, is added and combined as a single (composite) Model. With this, it always recalculates the SBG when the parameters of the assigned peak changes as well. For the fit itself a loop was written, that does not fit the complete first spectrum and then goes to the next one with derived fit parameters but first fits all of the

1st peaks of all spectra and then fits to the second peaks. Hereby another benefit of *lmfit* comes into play. The parameters of all peaks can be linked via expression (*expr*) commands. Therefore, the shapes of all corresponding peaks can be set to be the same, as well as the spin-orbit splitting to the literature values. This was done for all the oxidation states Cu(I), Cu(II), Ni(II), Ni(III) and loss features (Cu $2p_a$). The O KLL shape was left free.

Used software

To subtract the K_α-satellite peaks [Mou95] from each spectrum, a routine in the *Unifit2016* [Uni16] software was used.

In the *integration* approach a linear background from the spectra was subtracted using a script written in Python 3.8 [Pyt20].

To approximate the inelastic mean free path (IMFP) for the sample the respectively values were taken from the QUASES code [QUA19]. For the ratio dependent calculation see S.I.: *IMFP and Composition Determination*.

To determine the linear approximations of the 6th –order polynomial function, the software *fityk 0.9.4* [fit19] was used.

The fitting with the "active" Shirley BG was done using a fitting routine written in *Python 3.8* [Pyt20] (see S.I.: *Python script*).

References

[Cas06]: “Peak Fitting in XPS”, CasaXPS Copyright © Casa Software Ldt. www.casaxps.com (last checked May 2021) http://www.casaxps.com/help_manual/manual_updates/peak_fitting_in_xps.pdf

[fit19]: fityk 0.9.4 (2019) https://fityk.nieto.pl/

[HZB21]: HZB EMIL: SISSY PGM working range (last checked June 2021) https://www.helmholtz-berlin.de/pubbin/igama_output?modus=datei&did=917

[Lev44]: Levenberg, “A method for the solution of certain non-linear problems in leas squares”, (1994), *Quart. Appl. Math.* **2**, 164-168, DOI: 10.1090/qam/10666

[Mar63]: Marquardt, “An algorithm for least-squares estimation of nonlinear parameters” (1963), J. Soc. Indust. Appl. Math, 11 (2), DOI: 10.1088/0370-1328/73/5/310

[New14]: Newville et al., LMFIT: Non-Linear Least-Squares Minimization and Curve-Fitting for Python (version 0.8.0) (2014), *Zenodo,* DOI: 10.5281/zenodo.11813, (last checked May 2021)

[Pyt20]: Python Software Foundation. Python Language Reference, version 3.8, http://www.python.org, (2020)

[QUA19]: QUASES-IMFP-TPP2m Ver. 3.0, Downloaded 2019, http://www.quases.com/products/quases-imfp-tpp2m/

[SPE18]: SPECS Surface Nano Analysis GmbH, “User Manual XR 50-X-Ray Source, Version 3.2” (2018)

[Trz06]: Trzhaskovskaya et al.: (2006) *Atomic Data and Nuclear Data Tables* 92, 245-304

[Uni16]: Hesse, “Unifit2016“ (2020), *Universität Leibzig*, https://home.uni-leipzig.de/unifit/
